# Acute Pulmonary Embolism Following Baker’s Cyst Excision – A Life Threatening Complication: A Case Report

**DOI:** 10.5704/MOJ.2307.012

**Published:** 2023-07

**Authors:** V Senthil

**Affiliations:** Department of Orthopaedics, Government Royapettah Hospital, Chennai, India

**Keywords:** pulmonary embolism, Baker’s cyst, deep vein thrombosis, open Baker’s cyst excision

## Abstract

A 55-year-old women was diagnosed with Baker’s cyst and underwent open Baker’s cyst excision. She had developed acute pulmonary embolism in the post-operative period. Our case report is to emphasise the sub-clinical concomitant deep vein thrombosis with Baker’s cyst. Such a fatal complication has not been reported in literature and preventive measures of pre-operative venous Doppler and post-operative thrombo-prophylaxis can prevent them.

## Introduction

Baker’s cyst is considered as a valvular opening of posterior capsule deep to medial head of gastrocnemius. It is a synovial cyst in the popliteal region^1^. Baker’s cyst may compress the popliteal vein and causing deep vein thrombosis which appear sub-clinical in the patient^2^. We report a case of pulmonary embolism in an old woman following Baker cyst excision in the post-operative period. In this case report, we want to bring out the importance of Baker’s cyst as a causative factor of asymptomatic deep vein thrombosis leading to fatal thrombo-embolism causing death.

## Case Report

A 55-year-old female had presented with pain and swelling on the back of left knee for 6 months. Pain was insidious and progressive and increased in doing normal activities like climbing stairs, walking normal distances and sitting cross legged. Pain didn’t improve with anti-inflammatory and pain medications. The swelling was insidious onset, gradual progression with range of movements being 0°–120° with pain in terminal flexion. No history of trauma, fever, or constitutional symptoms. Patient was planned for Baker’s cyst excision in view of continuing pain and discomfort over the back side of knee despite being on medications for six months.

Patient was hypertensive and on Tab atenolol 25mg night dose for 10 years. On general physical examination patient was moderately built and nourished, Afebrile with stable vitals signs. Body mass index was 27.69kg/m^2^. Systemic examination detected no major structural or functional abnormalities in various systems.

**Fig 1:**
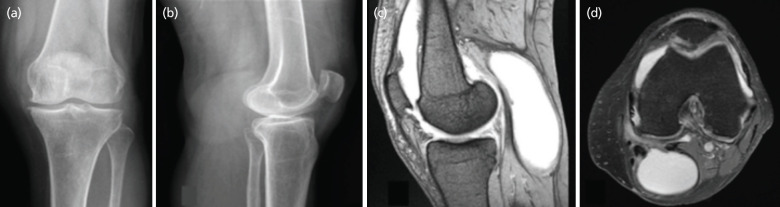
(a) Plain radiograph of the left knee AP, and (b) lateral view showing increased soft tissue density in the posterior part of knee. (c) Sagittal, and (d) axial view MRI showing Baker’s cyst extending between the semimembranous and medial head of gastrocnemius.

On local examination, diffuse swelling was present in the popliteal region. It was firm in consistency with ill-defined borders. Joint examination revealed mild joint effusion with medial joint line tenderness. Radiological examination revealed a dense shadow with well-defined border in the lateral view (Fig. 1a, 1b).

MRI revealed a Baker cyst (5x4.5x9.0cm) extending between the semi-membranous and the medial head of gastrocnemius with few thin internal septations along the inferior aspect of lesion (Fig. 1c, 1d). The patient was posted for surgery and pre-operative work was normal with hb-11.5g/dl, ESR-43mm/hr, CRP – 2.1mg/dl, HIV/HBSAg – non-reactive with normal RFT and coagulation profile.

Cardiac evaluation revealed mild left ventricular hypertrophy with mild MR. Valve motion abnormality, clot or pericardial effusion was absent. Ejection fraction was 61%. Pre-operative venous Doppler was not done because it wasn’t a part of the pre-operative protocol. Patient had no clinical indication of need for venous Doppler because clinical symptoms of deep vein thrombosis were not present.

**Fig 2:**
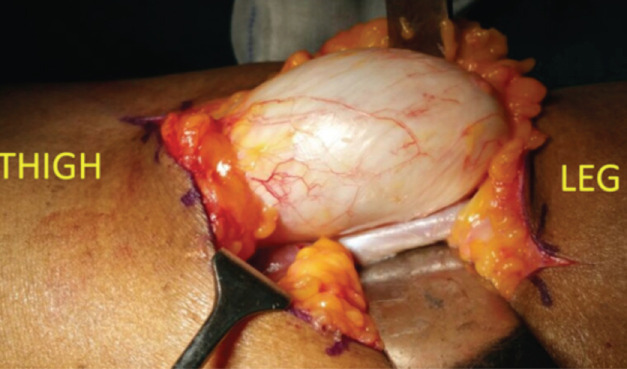
Clinical photograph showing the Baker’s cyst before complete excision.

Patient was posted for elective surgical procedure, under GA in prone position. Open Baker cyst excision was done under tourniquet control of 40 minutes duration (Fig. 2). Baker’s cyst was operated by the primary surgeon who is the author of this case report and was assisted by two final year ortho residents. Incision was made over the swelling and the skin and subcutaneous tissue were dissected. The swelling was delineated with the help of pushers made of gauze balls between the medial head of gastrocnemius and medial hamstring tendons (semi-membranous and semi-tendinous tendon). The swelling was removed in total by exposure of the stalk till the posterior capsule of knee. Gentle retraction with retractors was done to avoid any vascular injury. Post excision, tourniquet was deflated and compete haemostasis was achieved.

Intra-operative, patient had regular ectopic beats with an intra-operative blood pressure of 190/104. Her heart rate was on an average of 52bpm and saturation maintain at 100% with oxygen.

Baker’s cyst excision was uneventful in the immediate postoperative period with patient conscious, oriented and moving all four limbs. Post procedure compression bandage and knee immobiliser applied to the left lower limb. six hours post-surgery when patient was washing her face, she suddenly started sweating profusely and collapsed. Blood pressure was130/90mm/hg, SPO2 -68% at room air, GRBS was 128mg/dl. Patient was not responding to oral commands. She was shifted to ICU and was kept on ventilator support.

ECG was done suggested pulmonary embolism. ECG findings were sinus tachycardia with partial right ventricular strain S1Q3, subtle depression in lead 1, AVL, v5, v6 indicated that the patient was hypertensive.

**Fig 3:**
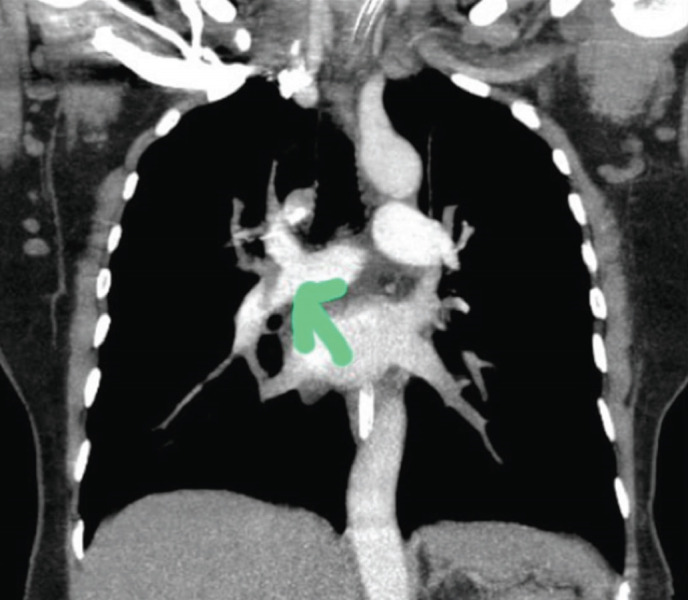
CT pulmonary image showing filling defect in the right pulmonary artery and branches (shown by green arrow).

CT pulmonary angiography showed filling defect in right pulmonary artery and branches suggesting pulmonary embolism (Fig. 3). D-dimer was 8.5mg and medications started were injection Tenecteplase 30mg IV stat and ultra-fractionated heparin 5000 units sub cutaneous 8th hourly.

CT brain showed multiple intra-axial hyper densities of blood attenuation noted in right frontal, parietal, temporal and occipital lobes suggesting haemorrhages and diffuse axonal injury. Clinically doll’s eye reflex, corneal reflex and gag reflex were absent. Spontaneous breathing was shallow. From the opinions of the neurosurgeon and cardio-thoracic vascular surgeon, conservative management was continued with no operative intervention.

Patient was monitored in ICU on ventilator. Soon the patient deteriorated and was declared brain dead after 48 hours of the index surgery. Prognosis was explained to the family members of the patient and was discharged against medical advice. On extubation patient had an episode of cardiac arrest. In-spite repeated attempts of revival, patient was declared dead. Autopsy was deferred due to non-acceptance by patient’s relatives.

## Discussion

Baker’s cyst is a popliteal cyst involving posterior gastrocnemius- semi-membranous bursa^1^. Majority of the Baker’s cyst present as asymptomatic mass in the popliteal fossa, rarely reported complications are dissection of the cyst mimicking deep vein thrombosis with swelling and stiffness in the posterior compartment. This is called as pseudo-thrombophlebitis syndrome^3,4^.

Association of Baker’s cyst with deep vein thrombosis is not uncommon and is reflected in the study by Simpson *et al*^4^, in which 36% of patients with DVT was complicated by the presence Baker’s cyst and 31% of patients with Baker’s cyst had DVT. The factors supporting this causation is the compression of vein by Baker’s cyst causing deep vein thrombosis which might be asymptomatic and additional surgical stress could have caused acute pulmonary embolism^5^.

Association of Baker’s cyst as the definitive cause of pulmonary embolism can’t be proved because of absence of pre-operative venous Doppler. Literature review has suggested that long standing Baker’s cyst is a predisposing factor of presence of sub-clinical deep vein thrombosis. Postoperative embolism to lungs and brain is the possible cause of death proved by CT investigation. Autopsy was not done in the absence of consent from patient relatives.

In our case, pulmonary embolism was confirmed by CT pulmonary angiogram. Physical findings were not present for DVT. Pulmonary embolism being a fatal complication. Venous Doppler scan of lower limb is recommended in patients with co-morbidities (DM, HTN, liver function with coagulopathies) as a part of pre-operative protocol. Limitation in our case report is the lack of pre-operative venous Doppler. Post-operative venous Doppler was deferred because CT pulmonary angiogram was diagnostic of pulmonary embolism.

Early detection of DVT with Baker’s cyst can prevent embolism. Presumptive anticoagulant prophylaxis can be started in immediate post-operative period to prevent fatal embolism. DVT prophylaxis was not started in our case, but we started static quadriceps and full weight bearing mobilisation with walker under post-operative femoral pain block.

Learning points in this case (1) association of Baker’s cyst with asymptomatic deep vein thrombosis to be considered as a risk factor for pulmonary embolism. (2) Pre-operative venous Doppler is needed to establish sub-clinical DVT to prevent life threatening complications and medico-legal issues following Baker’s cyst excision.
